# Expansion of effector and memory T cells is associated with increased survival in recurrent glioblastomas treated with dendritic cell immunotherapy

**DOI:** 10.1093/noajnl/vdz022

**Published:** 2019-08-20

**Authors:** Marica Eoli, Cristina Corbetta, Elena Anghileri, Natalia Di Ianni, Micaela Milani, Valeria Cuccarini, Silvia Musio, Rosina Paterra, Simona Frigerio, Sara Nava, Daniela Lisini, Sara Pessina, Luisa Maddaloni, Raffaella Lombardi, Maria Tardini, Paolo Ferroli, Francesco DiMeco, Maria Grazia Bruzzone, Carlo Antozzi, Bianca Pollo, Gaetano Finocchiaro, Serena Pellegatta

**Affiliations:** 1 Unit of Molecular Neuro-Oncology, Fondazione IRCCS Istituto Neurologico Carlo Besta, Milan, Italy; 2 Laboratory of Brain Tumor Immunotherapy, Fondazione IRCCS Istituto Neurologico Carlo Besta, Milan, Italy; 3 Unit of Neuro-Radiology, Fondazione IRCCS Istituto Neurologico Carlo Besta, Milan, Italy; 4 Cell Therapy Unit, Fondazione IRCCS Istituto Neurologico Carlo Besta, Milan, Italy; 5 3rd Neurology Unit and Skin Biopsy, Fondazione IRCCS Istituto Neurologico Carlo Besta, Milan, Italy; 6 Unit of Neurosurgery 2, Fondazione IRCCS Istituto Neurologico Carlo Besta, Milan, Italy; 7 Unit of Neurosurgery 1, Fondazione IRCCS Istituto Neurologico Carlo Besta, Milan, Italy; 8 Department of Pathophysiology and Transplantation, University of Milan, Milan, Italy; 9 Department of Neurological Surgery, Johns Hopkins Medical School, Baltimore, Maryland; 10 Unit of Neuro-Immunology, Fondazione IRCCS Istituto Neurologico Carlo Besta, Milan, Italy; 11 Unit of Neuropathology, Fondazione IRCCS Istituto Neurologico Carlo Besta, Milan, Italy

**Keywords:** dendritic cells, glioblastoma, immunotherapy, T-cell memory, tetanus toxoid

## Abstract

**Background:**

The efficacy of dendritic cell (DC) immunotherapy as a single therapeutic modality for the treatment of glioblastoma (GBM) patients remains limited. In this study, we evaluated in patients with GBM recurrence the immune-mediated effects of DC loaded with autologous tumor lysate combined with temozolomide (TMZ) or tetanus toxoid (TT).

**Methods:**

In the phase I-II clinical study DENDR2, 12 patients were treated with 5 DC vaccinations combined with dose-dense TMZ. Subsequently, in eight patients, here defined as Variant (V)-DENDR2, the vaccine site was preconditioned with TT 24 hours before DC vaccination and TMZ was avoided. As a survival endpoint for these studies, we considered overall survival 9 months (OS9) after second surgery. Patients were analyzed for the generation of effector, memory, and T helper immune response.

**Results:**

Four of 12 DENDR2 patients reached OS9, but all failed to show an immunological response. Five of eight V-DENDR2 patients (62%) reached OS9, and one patient is still alive (OS >30 months). A robust CD8^+^ T-cell activation and memory T-cell formation were observed in V-DENDR2 OS>9. Only in these patients, the vaccine-specific CD4^+^ T-cell activation (CD38^+^/HLA-DR^+^) was paralleled by an increase in TT-induced CD4^+^/CD38^low^/CD127^high^ memory T cells. Only V-DENDR2 patients showed the formation of a nodule at the DC injection site infiltrated by CCL3-expressing CD4^+^ T cells.

**Conclusions:**

TT preconditioning of the vaccine site and lack of TMZ could contribute to the efficacy of DC immunotherapy by inducing an effector response, memory, and helper T-cell generation.

**Importance of the Study**

There is no standard of care for recurrent/progressive glioblastoma (GBM), and their overall survival is approximately 9 months.

Dendritic cell (DC) immunotherapy is still showing promise, however, its efficacy as a single therapeutic modality is limited. Our previous evidence in primary GBM, suggested that temozolomide (TMZ) was ineffective as an adjuvant, due to its negative effects on T-cell activation and memory status generation. In our study, DC vaccinations without TMZ favor effector and memory T-cell response. Notably, the preconditioning of the vaccine site with tetanus toxoid induces a local infiltration of CCL3-expressing CD4^+^ T cells, a prerequisite for increasing DC migration to lymph nodes and consequently, antigen presentation, as previously demonstrated. The results strengthen the view that DC immunotherapy can be tailored to circumvent immunosuppression due to systemic, TMZ-based chemotherapy and thus activate the immune system, increasing survival expectations in the setting of GBM recurrence.

**Key points**

- In recurrent glioblastomas, dendritic cells and temozolomide fail to activate immunity.
- Tetanus toxoid induces a local infiltration of CCL3-expressing CD4^+^ T cells.
- Dendritic cells, tetanus toxoid, and lack of temozolomide activate T cells and improve survival.

Recurrence of glioblastoma (GBM), the most malignant primary brain tumor, does not have a standard treatment and is associated with a poor prognosis: median survival from recurrence is about 9 months.^1,2^

Immunotherapy (IT) has accomplished important prognostic improvements in different cancers and particularly in melanomas, mostly due to the treatment with checkpoint inhibitors.^3^ However, in patients affected by GBM evidence of meaningful clinical responses to checkpoint inhibitors is presently weak. Scarce and rare infiltration of T lymphocytes in the tumor, low mutational load, and the presence of a strong immune suppressive microenvironment, all characteristic of “cold” tumors, can partly explain resistance to checkpoint inhibitors as well as limitations in other strategies for GBM IT.^4^ Interestingly, two recent studies showed that neoadjuvant administration of PD1-blockade in recurrent GBM is associated to local immune responses and, in one of the two reports, prolonged survival.^5,6^

Dendritic cells (DCs), powerful antigen presenting cells, are an important tool for cancer IT.^7^ Recent data showing the T-cell cross talk for the deployment of anticancer T-cell immunity during checkpoint blockade supported the key role of DC.^8,9^ The efficacy of DC immunotherapy (DC-IT) as single therapeutic modality, however, is limited and rarely curative. This condition has generated considerable interest in combinatorial strategies.

Notably, some chemotherapeutic drugs may cause an immunogenic cell death, leading to some synergy with IT. Data on the impact of temozolomide (TMZ), which is part of the standard treatment of GBM,^10^ are partly contradictory. Some data suggested that TMZ-induced lymphopenia in combination with vaccination with an anti-EGFRvIII peptide may decrease the fraction of T helper cells^11^ and potentiate humoral responses.^12^ Other preclinical data and our own experience indicate that TMZ can impair the antitumor activity of CD8^+^ T cells.^13–16^

The efficiency of DC migration from the injection site to the lymph nodes (LNs) represents another critical aspect influencing the success of IT. It has been observed that less than 4%–5% of injected DCs can reach the LNs.^17^ Recent data from Mitchell et al.^18^ indicated that preconditioning the vaccine site with the recall antigen tetanus/diphtheria (Td) toxoid can induce a specific inflammatory immune response mediated by Td-specific CD4^+^ T cells and the production of CCL3, improving LN homing of DCs and, consequently, the efficacy of tumor-antigen-specific DCs.

Here, we report and compare the clinical and immunological data of recurrent GBM patients enrolled in DENDR2 study, in which DC were combined with a dose-dense TMZ, with Variant (V)-DENDR2 patients, in which recurrent GBM patients were treated with DCs after preconditioning of the injection site with tetanus toxoid (TT) recall in the absence of TMZ.

## Materials and Methods

### Clinical Study

Twenty patients have been considered overall.

The DENDR2 study (NCT04002804) was a phase I/II, two-stage Simon design, nonrandomized clinical study in which patients with recurrent GBM were treated with five injections of DCs and TMZ with a dose-dense schedule (TMZ 75 mg/m^2^ 3 weeks on and 1 week off for three cycles), previously proposed for treatment of GBM recurring after the standard treatment.^19^ A total of 16 patients were enrolled, but only 15 were evaluable for the efficacy endpoints (ie, they received at least three doses of DC vaccination). We restricted the patient analysis to 12 patients with IDH1 wild-type GBM. The prognostic role of IDH1-2 mutations was not fully appreciated when the DENDR2 protocol was approved and the presence of the mutations was not included in exclusion criteria. Eight patients satisfying the DENDR2 inclusion criteria were treated with DC vaccination combined with TT preconditioning and did not receive TMZ (variant DENDR2, V-DENDR2 for brevity). Safety, feasibility, and evidence of immune response were considered. The clinical protocol was approved by local and national regulatory authorities including Besta Ethical Committee, Istituto Superiore di Sanità (ISS) and AIFA (Italian Medicine Agency) and was sponsored by Fondazione IRCCS Istituto Neurologico Carlo Besta.

### Population and Treatment Protocol

Patients were enrolled in DENDR2 study after written informed consent. Inclusion criteria were the following: histologically proven GBM, age ≥18 and ≤70 years, no multifocal or subependymal diffusion of the tumor, residual tumor volume after surgery <10 ml, volume assessment by magnetic resonance imaging (MRI) after surgery, dexamethasone daily dose ≤4 mg during the 2 days prior to leukapheresis, Karnofsky Performance Status ≥70, availability of 0.8–1 g tissue for lysate preparation stored at −80 °C, absence of past or current autoimmune disease. After surgery, patients underwent leukapheresis. Monocyte-derived DC were loaded with whole tumor lysate and stored following Good Manufacturing Practice conditions.^20,21^ The first three vaccinations with DC were performed every 2 weeks (weeks 6–10). The fourth and fifth vaccinations were spaced 1 month (week 14 and 18, respectively). The first vaccine (I vacc) contained 20 million cells, second to fourth (II–IV vacc) contained 10 million cells, and the fifth vaccine (V vacc) contained 5 million cells. TMZ was administered for three cycles according to the schedule 21/28,^19^ before I, IV, and V vaccine. At each vaccine injection, clinical and immune monitoring were performed.

V-DENDR2 patients were treated with the same doses of DC vaccinations: preconditioning the vaccine site with the recall antigen (TT) (Anatetall; GSK Vaccine, 40 IU), intradermally injected the day before each DC vaccination. No TMZ was administered. Clinical, immunological, and radiological evaluations were performed with the same schedule, but a skin biopsy at the DC injection site was performed in all patients 3 days after the third vaccination.

### MRI and Response Evaluation

Patients underwent conventional contrast-enhanced MRI within 3 days after surgery, 2 days before the first vaccination, every 2 months, or in case of clinical indication. Tumor volumes were determined on the three-dimensional post gadolinium T1-weighted images by manually outlining the enhancing portion of the lesion in MRIcro (http://www.mricro.com) as previously described.^16^ To calculate the total enhancing volume of the tumor, the number of enhancing voxels was multiplied by the voxel size. Disease progression was defined according to RANO (Response Assessment in Neuro-Oncology) criteria.

### Immune Monitoring

Immune monitoring was performed on the whole blood of each patient before, during, and after DC vaccinations. REAfinity Recombinant Antibodies (Miltenyi Biotec) were used for the immune response characterizations. CD3, CD4, CD8, CD56, and CD45 were used to identify the T and NK cells. Antibodies for effector activation, memory status generation, and real-time PCR protocols are reported in Supplementary Methods.

Peripheral blood lymphocytes (PBLs) from 14 patients were cocultured in the presence of matched mature tumor lysate-pulsed DCs for 5 days in the presence of 30 U of Interleukin-2 (IL-2) (Roche). Interferon-gamma (IFN-γ) secretion was measured on the supernatants by ELISA assay (R&D Systems). IFN-γ expression was evaluated by intracellular staining with IFN-γ mAb (Miltenyi Biotec).

Acquisition of stained samples was performed using a MACSQuant (Miltenyi Biotec) flow cytometer, and data were analyzed using the Flowlogic software (version 7.2; Miltenyi Biotec).

### Immunohistochemistry

Cryostat frozen skin biopsies were sliced into 10-μm thick sections and fixed in acetone or 10% neutral buffered formalin. Sections were incubated with anti-CD4 (1:10; Dako) and anti-CCL3 (1:20; Thermo Fisher) antibodies overnight at 4 °C. Slides were counterstained with hematoxylin (Sigma-Aldrich), dehydrated, and mounted. After counterstaining with hematoxylin, sections were examined using a Leica microscope and analyses performed on two independent fields per section.

### Statistical Analyses

The Wilcoxon signed rank test was used to test the significance of differences between markers at various time points. All *P* values were two sided. The chi-square or Fisher exact tests were used to examine the differences in categorical variables between groups. For efficacy evaluation, only patients that underwent at least three vaccinations doses were considered. Overall survival (OS9) months from surgery for disease recurrence to death due to any cause or last follow-up (censored) was considered as a relevant endpoint. The log-rank test assessed differences in survival. All statistical analyses were performed using Prism 5.03 software.

## Results

### Patient Treatment and Survival

**Fig. 1.**
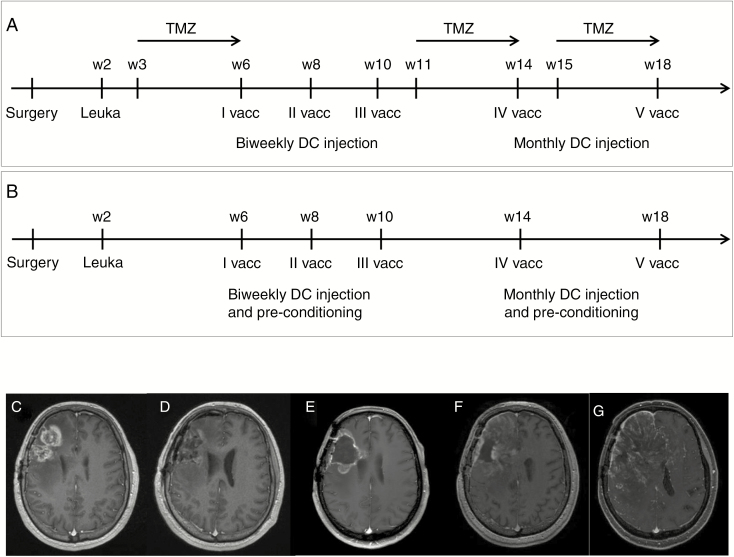
Treatment protocol and survival (**A** and **B**). Treatment schema for (A) DENDR2 patients and for (B) V-DENDR2 patients showing the timing before, during, and after the DC administrations. Leuka, leukapheresis; vacc, vaccine; TMZ, temozolomide; w, week. (**C**–**G**) Patient V-8. Contrast-enhanced T1-weighted image (wi) MRI (small box, pre-contrast T1-wi); (C) before surgery for recurrence: December 28, 2016; (D) after surgery for recurrence: December 30, 2016; (E) at the time of first DC vaccination: February 21, 2017; (F) 2 months after the fifth and last DC vaccination: July 17, 2017; (G) disease progression: December 12, 2017. DC, dendritic cell; MRI, magnetic resonance imaging.

Twenty patients with recurrent GBM enrolled in DENDR2 study were considered: 12 patients were treated with DC-IT concomitant with TMZ, and 8 patients, named (V)-DENDR2, were treated with DC-IT concomitant with TT in the absence of TMZ. We considered overall survival at 9 months (OS9) as a relevant survival endpoint based on recent phase II and III studies in recurrent GBM.^2,22^ The schedule of the treatment and clinical data are summarized in Fig. 1A and B, Supplementary Figure 1, and Table 1. The median interval between first and last surgery was 14.0 months (95% CI 11.2–25.6). Four patients completed all scheduled vaccinations, two patients discontinued treatment after four vaccinations, and six after three (Supplementary Figure 1). Five patients completed the TMZ schedule, five could be treated with two of three cycles, and two with one cycle only. Before surgery for recurrence, seven of these patients had completed the Stupp protocol.^10^ The median OS of DENDR2 patients was 7.4 months (95% CI 5.2–9.31) and OS9 was 33.3%. The median interval between last surgery and the first vaccine was 1.6 months (95% CI 1.4–1.78). All patients experienced death during the follow-up due to tumor progression. At the time of the first vaccination, the median tumor volume was 7.6 ml. In three patients (Pts 11, 16, and 17), disease progression occurred before starting the IT (Supplementary Table 1). At first vaccination the median dexamethasone dosage was 4 mg (mean: 3.6, range 0–6 mg). Four DENDR2 patients were at second recurrence when enrolled in the study (Pts 13, 17, 19, and 25).

**Table 1.** Patient characteristics

Patient	Age/gender	KPS	No. of TMZ cycles after second surgery	No. of vaccinations (total *n* = 5)	MGMT (Met ≥ 0.1)	Immune response^a^	OS (mos)	OS (mos) from diagnosis
11	66/M	90	2	5/5	M (8.370)	No	9.5	38.6
13	61/F	70	2	3/5	NA	No	7.4	23.8
16	57/F	60	2	3/5	U (0.095)	No	4.7	20.2
17	61/M	60	1	3/5	M (12.894)	No	4.9	18.1
18	54/M	90	3	4/5	U^b^	No	7.7	19.6
19	58/M	90	1	3/5	U (0.017)	No	11.5	23.4
20	68/F	70	2	3/5	U (0.066)	No	5.2	13.7
22	45/F	80	2	3/5	M (0.730)	No	5.2	35.6
23	60/M	70	3	4/5	U (0.060)	No	7.4	23.8
24	51/M	80	3	5/5	U (0.000)	No	7.5	22.6
25	42/M	80	3	5/5	M (0.390)	No	16.8	44.8
28	54/F	80	3	5/5	U (0.020)	No	9.3	29.2
V-1	44/M	70	none	4/4	M (0.320)	Yes	9.3	35.9
V-2	56/M	100	none	3/5	M (1.560)	No	5.8	21.4
V-3	39/M	90	none	3/5	U (0.010)	No	7.2	25.7
V-4	56/M	90	none	5/5	M (0.130)	Yes	11.1	27.8
V-5	61/M	70	none	5/5	U (0.000)	No	6.5	19.2
V-6	34/F	100	none	5/5	U (0.050)	Yes	>30.0	>30.0
V-7	69/F	80	none	5/5	M (2.120)	Yes	9.1	24.9
V-8	44/M	100	none	5/5	U (0.000)	Yes	14.2	22.9

KPS, Karnofsky Performance Score; M, methylated; MGMT, O^6^-methylguanine-DNA methyltransferase; mos, months; OS: overall survival; TMZ, temozolomide; U, unmethylated.

^a^Significant activation of T-cell response evaluated as V/B ratio >1.05.

^b^Immunohistochemistry analysis.

Hypermethylation of the O^6^-methylguanine-DNA methyltransferase (MGMT) promoter, evaluated by methylation-specific PCR,^23^ was detected in 4 of the 11 patients with enough DNA available for the analysis: two of them were patients surviving more than 9 months (OS>9). Two patients did not receive active treatment after progression (Pts 16 and 17), four were treated with bevacizumab, eight with chemotherapy, and one with chemotherapy and radiotherapy (RT) (Supplementary Table 2).

Five V-DENDR2 patients completed all scheduled vaccinations: patient V-1 discontinued treatment after four vaccinations and patients V-2 and V-3 suspended the treatment after the third vaccination due to clinical and radiological worsening but restarted the vaccinations after 5 and 2 months, respectively, when their condition improved (Supplementary Figure 1). After progression, three patients were treated with two cycles of PCV (procarbazine, CCNU, and vincristine) whereas the others received no further treatment (Supplementary Table 2). At the time of first vaccination the median tumor volume was 14.6 ml. In five patients (Pts V-1, V-2, V-3, V-6, V-7), tumor progression occurred before the first vaccination (Supplementary Table 1). When DC-IT started the median steroid dosage was 2 mg (mean: 2, range 0–4 mg) (Supplementary Table 1).

After a median follow-up of 9.2 months, one of the eight patients was alive, six died for tumor progression, and one for pulmonary embolism. Hypermethylation of the MGMT promoter was detected in four of the eight patients: two of them survived longer than 9 months. IDH1-2 were absent in all patients. The median OS was 9.2 (95% CI 5.2–9.31) months and OS9 was reached by 62.5% of the patients (Table 1). An exemplificative MRI of one V-DENDR2 (V-8) is displayed in Fig. 1C–G.

### Treatments in Both DENDR2 and V-DENDR2 Patients Are Safe and Well Tolerated

As reported in Supplementary Table 2, adverse events in DENDR2 included transient neurological worsening (possibly IT-related) and hematological toxicities (TMZ-related). Five V-DENDR2 patients showed neurological worsening after the third vaccination, one after the last vaccination, and two showed no symptoms. Seizures occurred in three cases, headache and confusion in patient V-2, and pneumonia in V-3, all related to the tumor progression. Weak skin reactions were recorded at the injection site after TT administration. Reactions after DC injection were characterized by skin redness and/or thickening that were weak in three patients (V-1, V-2 and V-5) and stronger in other three (V-3, V-4, and V-6).

### T-Cell Count and IFN-γ Secretion Strongly Increase in V-DENDR2 OS>9 Patients

**Fig. 2.**
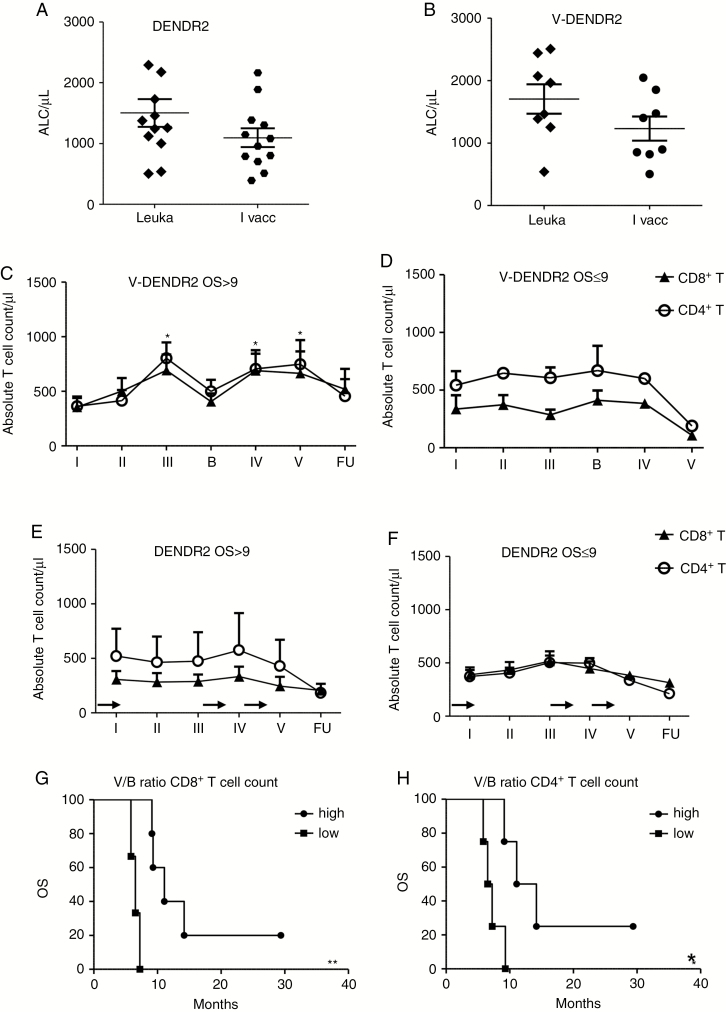
Absolute T-cell counts before and after treatment (**A**–**F**). (A and B) Absolute lymphocyte counts (ALCs) in the peripheral blood of patients at the time of the leukapheresis (leuka) and at the time of the first vaccination (I vacc), after the first cycle of TMZ administration, in DENDR2 patients (A); at leuka, at the time of TT preconditioning (I vacc) in V-DENDR2 patients (B). Data are presented as mean ± SD; (C–F) Time course of CD8^+^ and CD4^+^ absolute counts of V-DENDR2 OS>9 (C) and OS≤9 (D) patients over the treatment, including the time of the skin biopsy [B], (*P = .02 at III, *P* < .05 at IV, *P* = .04 at V vs. I vacc, where the count was revealed at the time of TT preconditioning; Fisher’s exact test *P* = .01), and of DENDR2 OS>9 (E) and OS≤9 (F) patients over the treatment. The arrows on *x*-axis indicate the TMZ administrations. Data are presented as mean ± SEM; (G and H) Kaplan–Meier curves showing the correlation between the V/B ratio of CD8^+^ and CD4^+^ T cells and OS per V-DENDR2 patients. OS, overall survival; TMZ, temozolomide; TT, tetanus toxoid.

We assessed patient immune responses considering the count and frequency of PBLs before treatment, at each vaccination, and after IT. We previously demonstrated that RT-TMZ treatment affected on absolute lymphocyte count (ALC) in DENDR1 patients, inducing significant lymphopenia.^16^ In DENDR2 at leukapheresis we observed a basal ALC > 1000 cells/ml in 9 of 10 patients (1504.4/ml ± 792.0/ml, mean ± SD), that decreased after TMZ treatment (1096.5/ml ± 530.4/ml, mean ± SD, *P* = .1) (Fig. 2A). In V-DENDR2, ALCs were 1704.6/ml ± 666.0/ml at leukapheresis and decreased to 1232.0/ml ± 546.7/ml (*P* = .1) at first vaccine (Fig. 2B).

The absolute count of both CD8^+^ and CD4^+^ T cells increased significantly after second, third, and fourth vaccination in V-DENDR2 OS>9 cases only (Fig. 2C). Neither V-DENDR2 OS≤9 nor DENDR2 patients showed a positive modulation of T-cell counts (Fig. 2D–F). Notably, V/B ratios (ratio of the mean of vaccination/baseline values) higher than 1.05 and 1.1 for CD8^+^ and CD4^+^ T-cell counts, respectively, were associated with prolonged OS (median OS 11.1 months vs. 6.5 months, *P* = .004; median OS 12.6 months vs. 6.8 months, *P* = .03) (Fig. 2G and H).

**Fig. 3.**
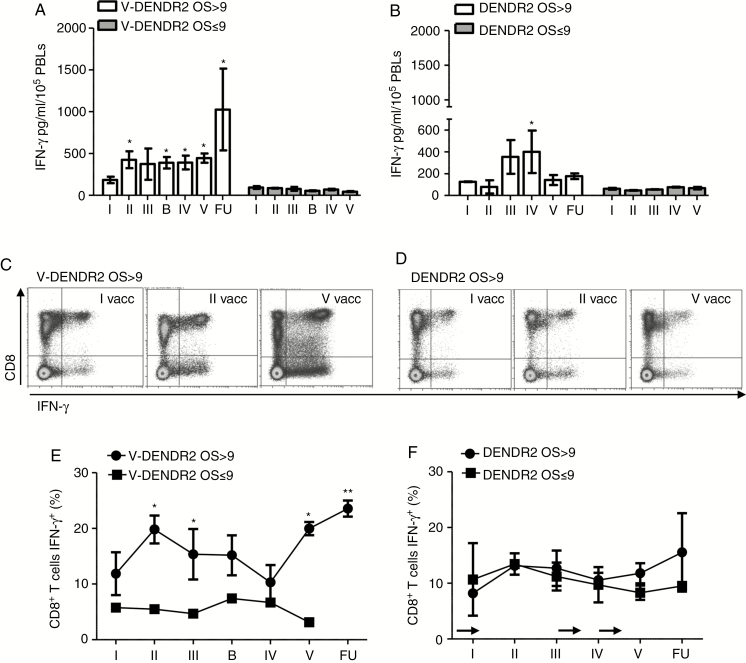
Characterization of antitumor immune response and memory formation. (**A** and **B**) Time course of IFN-γ secretion by PBLs cocultured for 5 days in the presence of matched autologous loaded mature DCs from V-DENDR2 (3 OS>9 and 3 OS≤9) (A) and from DENDR2 (4 OS>9 and 4 OS≤9) (B), over the treatment (including the time of biopsy [B] in V-DENDR2 patients and follow-up [FU] when performed). (**C** and **D**) Representative dot plots showing the CD8^+^ T-cell positivity for IFN-γ in V-DENDR2 OS>9 (C) and for DENDR2>OS9 (D). (**E** and **F**) Kinetics of the frequency of CD8^+^ T-cell-expressing IFN-γ assessed by flow cytometry of V-DENDR2 (E) and DENDR2 patients (F) (*P < .01, **P < .005 vs. I vaccination; Mann–Whitney test). The arrows indicate the TMZ administrations. Data are presented as mean ± SEM. DC, dendritic cell; OS, overall survival; PBL, peripheral blood lymphocyte; TMZ, temozolomide.

To evaluate the specificity of immune responses we cocultured available PBLs (14 patients, 8 DENDR2, and 6 V-DENDR2) with matched mature DC pulsed with autologous tumor lysate. IFN-γ production measured by ELISA increased in V-DENDR2 OS>9, but not OS≤9, with a significant increase at second vaccination that was maintained until the end of treatment (Fig. 3A). PBLs from two DENDR2 patients OS>9 (Pts 25 and 28) contributed to the significant increase of IFN-γ at fourth vaccination only (Fig. 3B).

The most stringent negative control, immature DCs, was not available for these experiments as the DENDR2 protocol did not include the storage of frozen immature DC; however, the absence of PBL activation with DC from nonresponders argues against their unspecific activation.

Detection of IFN-γ was also assessed by flow cytometry as intracellular expression in CD8^+^ and CD4^+^ T cells after phorbol 12-myristate 13-acetate (PMA)/ionomycin stimulation (Fig. 3C–F). A significant increase of CD8^+^ T-cell-expressing IFN-γ was observed in V-DENDR2 OS>9 at second vaccination compared to the I vaccination considered as the baseline (19.8 ± 5.5 vs. 11.8 ± 8.6, *P* < .05) (Fig. 3C–E). A contraction phase was present between the third and fourth vaccination and a rapid, second increase at fifth vaccination and at follow-up (19.9 ± 2.6 and 23.6 ± 3.2, respectively, *P* < .05 compared to the baseline), in line with of IFN-γ production after DC stimulation. A weaker response was observed for CD4^+^ T cells (Supplementary Figure 2A).

In V-DENDR2 OS≤9 and in all DENDR2 patients T cells did not show any activation (Fig. 3D–F; Supplementary Figure 2B). A significant NK cell response was only detected in V-DENDR2 OS>9 patients at earlier time points (Supplementary Figure 2C and D).

We also investigated the transition of effector T cells toward memory formation, by characterizing CD8^+^ and CD4^+^ T cells based on their expression of the antigen-experienced, effector T-cell marker KLRG1^24^ (Supplementary Figure 3A-F). CD8^+^ T effector cells coexpressing high levels of KLRG1 and IFN-γshowed a significant expansion and contraction phase in V-DENDR2 OS>9 only (Supplementary Figure 3A and C). In V-DENDR2 OS≤9, CD8^+^ IFN-γ ^+^ T cells retained high expression of KLRG1, precluding the formation of long-lasting T-cell memory (Supplementary Figure 3C). No significant increase of KLRG1^+^ T cells was detected in DENDR2 patients, indicating the lack of active and functional effector T cells (Supplementary Figure 3B, D, and F). Effector to memory transition of CD4^+^ T cells was weaker than the CD8^+^ T-cell response, decreasing slowly over time (Supplementary Figure 3E and F).

Real-time PCR revealed a predominant expression of EOMES and ID3 genes, indicating an enrichment for T central memory (Tcm), exclusively in V-DENDR2 OS>9 and during the contraction phase (Supplementary Figure 3G). At the same time points, V-DENDR2 OS≤9 showed an increased expression of the T effector memory (Tem)-associated genes T-BET and/or PRDM1 (Supplementary Figure 3G). No significant differences in memory composition were found in DENDR2 patients (Supplementary Figure 3H).

Overall, we can conclude that CD8^+^ T cells differentiate into long-lasting memory T-cell-expressing IFN-γ in V-DENDR2 OS>9 only.

### Activated Vaccine-Specific and TT-Induced CD4^+^ T Cells Are Identified in V-DENDR2 OS>9 Only

**Fig. 4.**
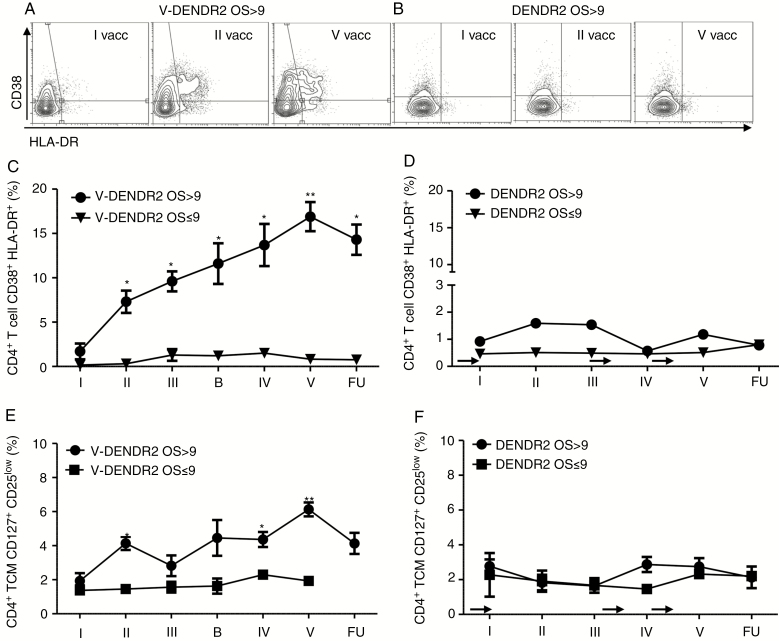
(**A** and **B**) Kinetics of the frequency of CD38^+^/HLA-DR^+^-activated cells evaluated in CD45/CD3/CD4^+^ T cells, after in vitro restimulation, in V-DENDR2 (A) (*P < .01, ** *P* < .005 vs. I vaccination; Mann-Whitney test) and DENDR2 patients (B); (**C** and **D**) Time course of CD4^+^ CD127^+^ CD25^low^ T-cells analysis in V-DENDR2 (C) (*P < .01, **P < .005 vs. I vaccination; Mann–Whitney test) and DENDR2 patients (D). The arrows indicate the TMZ administrations. TMZ, temozolomide.

Activation status of CD4+ T cells was investigated by analyzing the expression of specific markers including CD38 and HLA-DR (Fig. 4A and D).^25^ The double positive subpopulation increased during the treatment in V-DENDR2 OS>9 only (Fig. 4A and C). A rapid increase was observed immediately after the first DC vaccination (at the time of the second vaccination), persisting over time. The presence of this specific subset of activated/inducer T cells was undetectable in both V-DENDR2 OS≤9 (Fig. 4C) and DENDR2 patients (Fig. 4B and D).

We also tried to distinguish vaccine-specific from TT recall-induced CD4^+^ T helper memory cells. We first analyzed the memory T-cell subsets by assessing the memory markers CD45RA and CCR7.^26^ We identified the CD45RA^−^ CCR7^−^ as Tem and CD45RA^−^ CCR7^+^ as Tcm. A CD4^+^ CD38^+/high^ IFN-γ ^+^ T-cell subset (mainly CCR7^−^, consistent with vaccine-induced Tem), was expanded in V-DENDR2 OS>9 (Supplementary Figure 4A and C), and a CD4^+^ CD38^+/low^ IFN-γ ^+^ T-cell response (predominantly CCR7^+^, consistent with Tcm), displayed similar kinetics (Fig. 4A). Both these subsets were absent at the baseline (first vaccination), displayed a rapid activation after the second vaccination and later.

In V-DENDR2 OS≤9, these two subsets of CD4^+^ T cells were absent at the baseline (Supplementary Figure 4D). Only the CD38^+/high^ T cells significantly increased at the third vaccination and returned rapidly to baseline values. These subsets were not detectable in DENDR2 patients (Supplementary Figure 4B, E, and F).

In V-DENDR2 OS>9 only, the CD4^+^ Tcm cells also expressed high levels of CD127, the receptor of IL-7 involved in memory T-cell survival and persistence, and low levels of CD25^27^ (Fig. 4E). Overall, the expansion of vaccine-specific activated memory T cells was accompanied by an increase of a bystander CD4^+^ T helper memory cells defined as CD38^+/low^ and CD127^+^/CD25^low^.^25^ Modulation of CD4^+^ T helper memory cells was absent in both V-DENDR2 OS≤9 and DENDR2 patients (Fig. 4E and F).

### Skin Thickenings at the Tetanus Toxoid Injection Site Are Infiltrated by CCL3-Expressing CD4^+^ T Cells

To confirm the contribution of TT preconditioning in enhancing the efficacy of DC vaccinations, we monitored the appearance of a local reaction during the treatment.

**Fig. 5.**
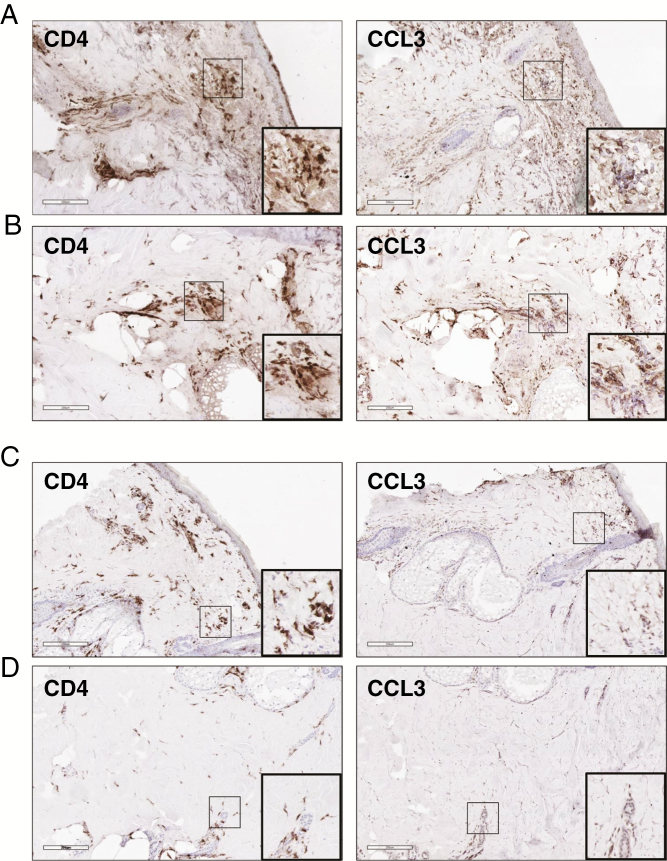
Investigation of the CCL3-expressing CD4^+^ T cells in skin biopsies. (**A** and **B**) Two V-DENDR2 and two control patients (**C** and **D**) have been investigated, and representative images are displayed. (A and B) Rectangles indicating the same areas in adjacent sections of the skin biopsies show a robust dermal infiltration of CD4^+^ T cells (left panels) expressing CCL3 (right panels). (C and D) In the control skin biopsies, the immunohistochemistry reveal a moderate (C) and low (D) infiltration of CD4^+^ T cells and negative for CCL3. Scale bar, 200 μm.

In the absence of TT preconditioning (ie, in DENDR2 patients), skin reactions to DC injections were absent. In V-DENDR2 patients we observed the formation of granulomas at the DC injection site, appearing as localized thickenings of different sizes, that we removed by skin biopsy in four patients after the third vaccination. As controls, we used the thickening that appeared at the DC injection site in a minority of patients enrolled in the DENDR1 study.^16^ Staining for CD4 and CCL3 was performed on adjacent sections of the skin biopsies derived from two V-DENDR2 OS>9 and two DENDR1 responders, as controls (Fig. 5A–D). The V-DENDR2 skin biopsies were characterized by dermal infiltration of CD4 and CCL3 positive cells (Fig. 5A and B). In DENDR1 patients the CD4 positive cells were preferentially distributed near the upper layer of the dermis, and CCL3 positive cells were not found (Fig. 5C and D).

## Discussion

The improvement of combinatorial strategies to increase IT efficacy is an ongoing challenge. A potential synergy between IT and chemotherapy has been reported due to the influence of some chemotherapeutic agents on tumor-specific immune responses, either by inducing immunogenic cell death of tumor cells or by modulating key cells for immune suppression or activation.^11,12,28^

In the clinical study on newly diagnosed GBM that is active at our institution, vaccinations with DCs loaded with whole tumor lysate were combined with TMZ chemotherapy administered as an adjuvant treatment in the context of the Stupp regimen.^10^ We obtained encouraging results in a fraction of these patients (45%), associated with a rise of active NK cells in peripheral blood; however, the contribution of CD8^+^ T cells to antitumor activity and the memory status generation was negligible. TMZ toxicity, that was limited in NK cells where the drug transporter ABCC3 was more expressed that in CD8^+^ T cells, may partly explain our findings.^16^

Our previous evidence in primary GBM indicated that the activation of CD8^+^ T cells was not compromised by the administration of TMZ combined with RT. However, a rapid and persistent depletion of CD8^+^ T cells was observed after the fourth vaccination when DC vaccines were administrated in combination with TMZ.^16^ Interestingly, a similar kinetics showing a decline of vaccine response after the fourth vaccination was observed in patients treated with cytomegalovirus pp65-targetd vaccinations as reported by Batich et al.^29^, supporting a potential negative effect induced by the continuation of concomitant TMZ administration.

Thus, our experience with DC-IT indicated that systemic administration of TMZ in standard treatment can limit the antitumor immune response of CD8^+^ T cells and their long-lasting response. Interestingly, preclinical data recently indicated that standard, but not metronomic dose, TMZ increased exhaustion markers in tumor infiltrating lymphocytes.^30^

Interestingly, systemic, intraperitoneal delivery of BCNU (bis-chloroethyl nitrosurea), frequently used as second line chemotherapy in recurrent GBM, did not synergize with IT,^31^ whereas the local delivery of BCNU “wafers” into the tumor cavity significantly enhanced the antitumor activity of checkpoint inhibitors.

In this report, we have compared two different strategies of DC-IT for recurrent GBM. In DENDR2, where DC vaccinations were combined with dose-dense TMZ, we were unable to detect any significant activation of immune responses and survival advantage. Only four DENDR2 patients survived more than 9 months with weak correlations with peripheral immune activation. Two of them (Pt 25 and Pt 28) showed a significant secretion of IFN-γ at fourth vaccination only, detected by ELISA in the PBLs and matched DC cocultures, and a minor increase of NK cell frequency between the second and the fourth vaccinations. Overall DENDR2 patients failed to show the marked activation of NK cells observed in DENDR1 patients.^16^ We hypothesize that exposure of NK cells to TMZ concentrations higher than in DENDR1 may have caused their exhaustion. Indeed, NK cells are susceptible to become exhausted and unable to produce IFN-γ and exert cytotoxic activity.^32^ Furthermore, NK cells can acquire an exhausted phenotype during tumor progression, through the action of the checkpoint receptor TIGIT.^33^

On the basis of clinical and immunological negative data from DENDR2 patients, we treated eight patients named V-DENDR2 where TMZ was avoided. We also considered the ability of DCs to migrate to LNs as another critical aspect impinging on the efficacy of IT. Recently, Mitchell et al. have demonstrated that antitumor activity associated with DC vaccinations can be increased by administering TT as a preconditioning step.^18^ As virtually all people have been vaccinated against tetanus, the recall antigen can attract locally CD4^+^ T cells that release the CCL3 chemokine, upregulating the expression of CCL21. This chemokine improved DC homing to the LNs and was associated with evidence of a significant clinical advantage in a murine model of GBM and in a limited number of patients.^18^

The V-DENDR2 recurrent GBM patients received TT the day before DC injection and no TMZ. Increased survival and specific effector and helper immune responses were found in five of these patients who survived longer than 9 months (“OS>9”). In two of them (V-1 and V-4), DC IT was temporarily discontinued but proposed again when the clinical and radiological pictures improved, suggesting that their previous symptoms were due to pseudo progression, likely caused by antitumor immune responses.

The activation of antigen-specific CD4^+^ T cells was investigated by evaluating the coexpression of CD38 and HLA-DR. On the basis of the evidence that CD4^+^ T cells respond to TT,^34^ we examined the expansion of TT-specific CD4^+^ T cells, looking for the subset expressing low levels of CD38 and positive for IFN-γ and high expression of CD38 and CD127 markers.^25^ Given the fact that these CD38^+^ T cells are a subset of memory T cells, we found a low frequency of CD38^+^ T cells. Their modulation was only evident in V-DENDR2 OS>9.

The increased frequency of CD4^+^ T helper cells that are required to help CD8^+^ T-cell responses^35^ was associated with clinical advantage in five patients, who survived longer than 9 months. Immunological parameters, including ALC, V/B ratio, and OS were not significantly affected by steroid dosage and tumor volume at the first vaccination.

Also, an infiltration of cells positive for CD4 and CCL3 was visible in skin biopsies obtained after the sequence of TT conditioning and DC injection, in good agreement with data from Mitchell et al..^18,36^

Our data cannot dissect what fraction of the immune response and corresponding survival advantage can be ascribed to the use of TT or the lack of TMZ. However, these findings encourage larger studies on GBM patients in the recurrent setting using DC after preconditioning of the injection site (https://clinicaltrials.gov/ct2/results?cond=glioma&term=tetanus&cntry=&state=&city=&dist). Result confirmation may open the way to further studies attempting to increase DC responses and other IT approaches by increasing neo-antigen presentation. Preclinical data suggest that combination with local chemotherapy,^31^ RT,^37^ and possibly high-intensity focused ultrasounds^38^ could be investigated. Recent clinical evidence may imply that neoadjuvant rather than adjuvant blockade of immune checkpoints creates a “warmer” environment in GBM facilitating immune responses orchestrated by DC.^5,6^

## Supplementary Material

vdz022_suppl_Supplementary_Methods
