# Supplementary material for: Expansion of effector and memory T cells is associated with increased survival in recurrent glioblastomas treated with dendritic cell immunotherapy
Source: Neurooncol Adv. 2019 Aug 20;1(1):vdz022. doi: 10.1093/noajnl/vdz022 (PMC7212883; doi:10.1093/noajnl/vdz022)
Supplement: vdz022_suppl_Supplementary_Methods [file vdz022_suppl_supplementary_methods.docx]

**Supplementary Figures and Tables**

**Expansion of effector and memory T cells is associated with increased survival in recurrent glioblastomas treated with dendritic cell immunotherapy**

Marica Eoli^1§^, Cristina Corbetta^1,2§^, Elena Anghileri^1§^, Natalia Di Ianni^1,2^, Micaela Milani^1,2^, Valeria Cuccarini^3^, Silvia Musio^1,2^, Rosina Paterra^1^, Simona Frigerio^4^, Sara Nava^4^, Daniela Lisini^4^, Sara Pessina^1,2^, Luisa Maddaloni^1^, Raffaella Lombardi^5^, Maria Tardini^1^, Paolo Ferroli^6^, Francesco DiMeco^7,8,9^, Maria Grazia Bruzzone^3^, Carlo Antozzi^10^, Bianca Pollo^11^, Gaetano Finocchiaro^1#^, Serena Pellegatta^#1,2^

*Supplementary data*

**Figure S1.** Patient treatment

(A, B) Schema of the patients receiving DC vaccines for DENDR2 (A) and V-DENDR2 patients (B). The number of the patients can change at every time point.

DENDR2 or V-DENDR2 patients surviving more than 9 months (OS>9) are highlighted in blue, DENDR2 or V-DENDR2 patients surviving less than 9 months (OS≤9) in red.

**Figure S2.** Characterization of effector response.

(A, B) Kinetics of frequency of CD4^+^ T cells expressing IFN-γ assessed by flow cytometry in V-DENDR2 (A) and DENDR2 patients (B) over the treatment (including the time of biopsy [B] in V-DENDR2 patients and follow-up [FU] when performed). (C, D) Kinetics of frequency of NK cells evaluated by flow cytometry in V-DENDR2 (C) and DENDR2 (D) patients over the treatment (including the time of biopsy in V-DENDR2 patients and follow-up when performed) (* P<0.01 and **P<0.005 vs first vaccine).

**Figure S3.** Characterization of effector to memory transition and T helper memory response.

(A, B) Exemplificative flow cytometry dot plots representing the double-positive KLRG1/IFN-γ cells gated in CD45/CD3/CD8+T cells in a V-DENDR2 OS>9 (A, Pt V-6) and in a DENDR2 OS>9 (B, Pt25); (C, D) Kinetics of the frequency of CD8^+^ T cells co-expressing KLRG1 and IFN-γ in V-DENDR2 (C), and DENDR2 patients (D) over the treatment (including the time of biopsy [B] in V-DENDR2 patients and follow-up [FU] when performed). A significant increase with respect to the I vaccination (I) set as the baseline, was found at second vaccination (6.4 ± 3.5% vs 0.9 ± 0.8%, respectively; P < 0.005). A rapid reduction started during the contraction phase (4.7 ± 2.7% vs second vaccination) and the memory phase at the follow-up (2.1 ± 1.8% vs second vaccination) (* P < 0.01 vs I vacc, underlined asterisk vs. II vacc; Mann-Whitney Test); (E, F) Kinetics of frequency of CD4^+^ T cells expressing KLRG1 and IFN-γ assessed by flow cytometry in V-DENDR2 (E) and DENDR2 (F) patients over the treatment (including the time of B in V-DENDR2 patients and FU when performed). (G, H) Bar graphs showing the relative expression of T central memory (EOMES, ID3) or T effector memory (TBET, PRDM1) associated genes in PBLs over the treatment in V- DENDR2 (G) and DENDR2 (H) patients, evaluated by real time PCR. The relative expression of genes was compared with that detected at the first vaccine (dotted line) (* P<0.01 and **P<0.005 vs first vaccine).

**Figure S4.** Characterization of CD4+ T cells potentially activated by TT recall

(A, B) Representative flow cytometry dot plots showing double-positive CD38/IFN-γ T cells gated on CD45/CD4 at first, second and fifth vaccine assessed by flow cytometry in V-DENDR2 (A) and DENDR2 (B) OS>9 patients. (C, D) Kinetics of the frequency of CD38^+/high^ and CD38^+/low^ expressing IFN-γ evaluated in CD45/CD3/CD4^+^ Tcm and Tem subset, after in vitro re-stimulation, in V-DENDR2 (C) OS>9 and (D) OS≤9. (V-DENDR2 OS>9. CD38^+/high^ cells: 3.4±0.5% vs 0.4±0.06%; CD38^+/low^ cells: 2.8 ± 0.4% vs 0.7 ± 0.3%, fourth vaccination vs baseline; P < 0.005 and P < 0.01, respectively; CD38^+/high^ cells: 2.8 ± 0.3% vs 0.4 ± 0.06%; CD38^+/low^ cells: 2.5 ± 0.7% vs 0.7 ± 0.3%, FU vs baseline, P < 0.001); (V-DENDR2. OS≤9: CD38+/high T cells: 2.1 ± 0.5% vs 0.3 ± 0.04%, third vaccination vs baseline) (* P < 0.01, ** P < 0.005 vs I vaccination; Mann-Whitney Test); (E, F) Kinetics of frequency of CD4^+^ T cells expressing CD38^high/low^ and IFN-γ assessed by flow cytometry in DENDR2 patients over the treatment (including the FU when performed).

*Supplementary Tables*

**Table S1. Steroids and tumor volume**

| Patient | Steroids at leukapheresis  (mg) | Steroids at I vaccine  (mg) | Tumor volume at  I vaccine (ml) |
| --- | --- | --- | --- |
| 11 | no | 4 | 19.4 |
| 13 | 4 | 6 | 9.8 |
| 16 | 4 | 3 | 48.9 |
| 17 | 2 | 4 | 23.7 |
| 18 | 4 | 4 | 4.3 |
| 19 | no | 1.85 | 0.8 |
| 20 | no | 6 | 5.2 |
| 22 | 8 | 4 | 8.4 |
| 23 | no | 4 | 7.5 |
| 24 | 6 | 4 | 9.8 |
| 25 | 4 | 2 | 0.8 |
| 28 | 4 | no | 5.6 |
| V-1 | 8 | 4 | 31.1 |
| V-2 | 4 | no | 61.0 |
| V-3 | 2 | 4 | 16.5 |
| V-4 | no | no | 2.5 |
| V-5 | 8 | 4 | 8.8 |
| V-6 | no | no | 1.6 |
| V-7 | no | no | 31.2 |
| V-8 | no | 4 | 12.8 |

**Table S2. Treatments after DC immunotherapy**

| Patient | Treatments after PD at the end of IT |
| --- | --- |
| 11 | Bevacizumab (2 cycles) |
| 13 | Bevacizumab (4 cycle) |
| 16 | No treatment |
| 17 | No treatment |
| 18 | FTM (3 cycles) |
| 19 | Bevacizumab (>10 cycles) + CCNU |
| 20 | CCNU (1 cycle) |
| 22 | CCNU |
| 23 | CCNU+ PCZ (2 cycles ) |
| 24 | Bevacizumab (3 cycles) |
| 25 | TMZ 21/28, from DENDR2 and on (tot 4 cycle); ETP |
| 28 | PCV /RT |
| V-1 | PCV (2 cycles) |
| V-2 | PCV (2 cycles) |
| V-3 | PCV (2 cycles) |
| V-4 | No treatment |
| V-5 | No treatment |
| V-6 | No treatment |
| V-7 | No treatment |
| V-8 | No treatment |

Abbreviations. DC: Dendritic cells; PD: progression disease; IT: Immunotherapy; FTM: fotemustine; CCNU: Lomustine; TMZ: temozolomide; PCZ: procarbazine; ETP: etoposide; PCV: Procarbazine, CCNU, and Vincristine; RT: radiotherapy

**Table S3. Adverse Events**

| **Adverse Events (AE)** | **No. of events** | | **Surgery-related** | **IT-related** | **TMZ-related** |
| --- | --- | --- | --- | --- | --- |
|  | **D2** | **V-D2** |  |  |  |
| **Brain edema** | 2 | 0 | NO | YES | NO |
| **Fever** | 1 | 0 | YES | NO | NO |
| **Nausea** | 1 | 2 | YES | NO | NO |
| **Vomiting** | 0 | 1 | NO | YES | NO |
| **Pain** | 2 | 0 | YES | NO | NO |
| **Asthenia** | 2 | 0 | NO | YES | YES |
| **Headache** | 1 | 3 | YES | NO | NO |
| **Seizure** | 1 | 3 | NO | YES | NO |
| **Neurosurgical wound complication** | 2 | 0 | YES | NO | NO |
| **Surgical site collection** | 2 | 0 | YES | NO | NO |
| **Dysphasia** | 0 | 2 | NO | YES | NO |
| **Dizziness** | 0 | 1 | NO | YES | NO |
| **Cognitive disturbance** | 0 | 2 | NO | YES | NO |
| **Hypostenia** | 0 | 3 | NO | YES | NO |
| **Intracranial bleeding** | 0 | 1 | NO | NO | NO |
| **Pulmonary embolism** | 0 | 1 | NO | NO | NO |
| **Pneumonia** | 0 | 1 | NO | NO | NO |
| **HEAMATOLOGICAL TOXICITIES:** |  |  |  |  |  |
| **Thrombocytopenia** | 2 | 0 | NO | NO | YES |
| **Lymphopenia** | 10 | 2 | NO | NO | YES |
| **Anemia** | 5 | 2 | YES | NO | YES |
| **Leucopenia** | 1 | 0 | NO | NO | YES |
| **Neutropenia** | 1 | 0 | NO | NO | YES |
| **Liver abnormality** | 1 | 0 | NO | NO | YES |
| **VACCINATION-SITE REACTIONS**  **Eythema** | 0 | 6 | NO | YES | NO |
| **Pruritus** | 0 | 0 | NO | YES | NO |
| **Pain and induration** | 0 | 5 | NO | YES | NO |

Abbreviations. IT: immunotherapy; TMZ: temozolomide; No.: numbers; D2: DENDR2; V-D2: Variant-DENDR2

*Supplementary Methods*

**Immune monitoring**

A total of 100 μL of whole blood were incubated with 10 μL of conjugated primary antibodies for 10 min at 4°C in the dark. The lysis of erythrocytes and the fixation of stained leukocytes were performed using the Uti-Lyse Erythrocyte Lysing Reagent (Dako) according to the manufacturer’s instructions for the “no wash” staining procedure.

Peripheral blood lymphocytes (PBLs) from each patient were frozen before, after each vaccination, at the follow-up (if performed) and at the time of the biopsy for V-DENDR2 patients. The memory status of CD8+ and CD4+ T cells was evaluated using anti-CD3, CD8, CD4, CD45RA and CCR7 mAbs (Miltenyi Biotec). Non-viable cells were discriminated by using the Viobility dye (Miltenyi Biotec). The effector and central memory were also investigated using anti-KLRG1, CD38, HLA-DR, CD127, CD25 mAb (Miltenyi Biotec). The expression of IFN-γ expression was assessed on thawed PBLs after restimulation with 50 ng/ml phorbol myristate acetate (PMA), 1 µg/ml Ionomycin and 10 µg/ml Brefeldin A for a total of 4 hours. Lymphocytes were fixed and permeabilized using the BD Cytofix/Cytoperm solution kit (BD Biosciences).

**Real time-PCR**

Total RNA was extracted from PBLs using Trizol (Life Technologies). Total RNA was reverse-transcribed using a High Capacity cDNA Synthesis KIT (Applied Biosystems-Life Technologies). The expression of EOMES, ID3, TBET, PRDM1 genes was detected by SYBR Green chemistry performed on ViiA7 Real-Time PCR system (Life Technologies), and normalized relative to β-actin. The RNA from the first vaccine was used as the calibrator for the calculation of fold expression levels with the ΔΔCt method.

Oligo Sequences:

EOMES FW: ATGGGTGACCTGTGGCAAAG; RV: TCCTGTCTCATCCAGTGGGA

ID3 FW: CAGCGCGTCATCGACTACAT; RV: TGACAAGTTCCGGAGTGAGC

TBET FW: CAAAGGATTCCGGGAGAACT; RV: TAGTGATCTCCCCCAAGGAA

PRDM1 FW: GTGTGGTATTGTCGGGACTTTG; RV: CAGTGCTCGGTTGCTTTAGAC

**Immunohistochemistry**

Endogenous peroxidase activity was quenched with 3% hydrogen peroxide in distilled water. Slides were treated with 1% bovine serum albumin (BSA) (Santa Cruz Biotechnology) and 5% normal goat serum in PBS containing 0.05% Triton X-100 (Sigma-Aldrich) and incubated in a closed humid chamber for 30 min at room temperature.

Staining was detected using the EnVision+ System-HRP–labeled polymer secondary antibodies for 1 hour at room temperature and then the chromogen DAB/substrate reagent (Dako). Slides were counterstained with hematoxylin (Sigma-Aldrich), dehydrated, and mounted.

**Statistical analyses**

The ratio of the mean (second to fifth) vaccination to baseline values (V/B ratio) of absolute count of CD8+ and CD4^+^ T cells for each patient was calculated, and the median of all of the observations was used as the cut-off value to separate patients into the “low” or “high” groups. Kaplan-Meier analysis was used to estimate OS.
